# The Ins and Outs of the BCCAo Model for Chronic Hypoperfusion: A Multimodal and Longitudinal MRI Approach

**DOI:** 10.1371/journal.pone.0074631

**Published:** 2013-09-18

**Authors:** Guadalupe Soria, Raúl Tudela, Ana Márquez-Martín, Lluïsa Camón, Dafnis Batalle, Emma Muñoz-Moreno, Elisenda Eixarch, Josep Puig, Salvador Pedraza, Elisabet Vila, Alberto Prats-Galino, Anna M. Planas

**Affiliations:** 1 Experimental 7 T MRI Unit, Institut d’Investigacions Biomèdiques August Pi i Sunyer (IDIBAPS), Barcelona, Spain; 2 Department of Brain Ischemia and Neurodegeneration, Institut d’Investigacions Biomèdiques de Barcelona (IIBB), Consejo Superior de Investigaciones Científicas (CSIC), Barcelona, Spain; 3 CIBER de Bioingeniería, Biomateriales y Nanomedicina (CIBER-BBN), Group of Biomedical Imaging of the University of Barcelona, Barcelona, Spain; 4 Departament de Farmacologia, Terapèutica i Toxicologia, Institut de Neurociències, Facultat de Medicina, Universitat Autònoma de Barcelona, Bellaterra, Spain; 5 Fetal and Perinatal Medicine Research Group, Institut d’Investigacions Biomediques August Pi i Sunyer (IDIBAPS), Barcelona, Spain; 6 IDI, Radiology Department, Hospital Universitario Dr. Josep Trueta. IDIBGI. Universitat de Girona, Girona, Spain; 7 Laboratory of Surgical Neuroanatomy (LSNA), Human Anatomy and Embryology Unit, Faculty of Medicine, Universitat de Barcelona, Barcelona, Spain; Massachusetts General Hospital/Harvard Medical School, United States of America

## Abstract

Cerebral hypoperfusion induced by bilateral common carotid artery occlusion (BCCAo) in rodents has been proposed as an experimental model of white matter damage and vascular dementia. However, the histopathological and behavioral alterations reported in this model are variable and a full characterization of the dynamic alterations is not available. Here we implemented a longitudinal multimodal magnetic resonance imaging (MRI) design, including time-of-flight angiography, high resolution T1-weighted images, T2 relaxometry mapping, diffusion tensor imaging, and cerebral blood flow measurements up to 12 weeks after BCCAo or sham-operation in Wistar rats. Changes in MRI were related to behavioral performance in executive function tasks and histopathological alterations in the same animals. MRI frequently (70%) showed various degrees of acute ischemic lesions, ranging from very small to large subcortical infarctions. Independently, delayed MRI changes were also apparent. The patterns of MRI alterations were related to either ischemic necrosis or gliosis. Progressive microstructural changes revealed by diffusion tensor imaging in white matter were confirmed by observation of myelinated fiber degeneration, including severe optic tract degeneration. The latter interfered with the visually cued learning paradigms used to test executive functions. Independently of brain damage, BCCAo induced progressive arteriogenesis in the vertebrobasilar tree, a process that was associated with blood flow recovery after 12 weeks. The structural alterations found in the basilar artery were compatible with compensatory adaptive changes driven by shear stress. In summary, BCCAo in rats induces specific signatures in multimodal MRI that are compatible with various types of histological lesion and with marked adaptive arteriogenesis.

## Introduction

Cerebrovascular pathology is involved in several forms of dementia. Vascular changes such as arterial stiffness, arteriolosclerosis, endothelial degeneration, and blood-brain barrier dysfunction are often associated with chronic cerebral hypoperfusion in disorders of the aging human brain [1]. On an etiological basis, several subtypes of vascular cognitive impairment have been proposed [2], the most prominent being multi-infarct dementia, mixed cortical and subcortical infarct dementia, small vessel disease, subcortical ischemic vascular disease, and CADASIL (Cerebral Autosomal Dominant Arteriopathy with Subcortical Infarcts and Leukoencephalopathy). Affecting deep penetrating arteries, small vessel disease causes lacunar infarcts and diffuse white matter lesions, and it is associated with vascular cognitive impairment [3]. Given the prevalence of subcortical ischemic vascular disease, there are considerable research efforts focus on this condition. Indeed, chronic hypoperfusion, for instance resulting from atherosclerosis, can lead to complete (lacunae) or incomplete stroke. The latter manifests as diffuse white matter lesions (leukoaraiosis) that typically affect the prefronto-subcortical circuits, which may explain some of the cognitive deficits often associated with executive functions.

In experimental animals, cerebral hypoperfusion accelerates cerebral amyloid angiopathy, promotes cortical microinfarcts in a mouse model [4], and induces cognitive deficits at an early stage of amyloid pathology in a transgenic mouse model of Alzheimer’s disease [5]. Bilateral common carotid artery occlusion (BCCAo) in rats has been proposed as a model of vascular dementia and has been widely used over recent years. However, the studies performed to date show variable results [6,7,8,9] because of the different structures addressed and the different time points and techniques used. Overall, brain damage is characterized by white matter lesions, with vacuolation of myelin, axonal damage, and demyelination in the corpus callosum, internal capsule, and caudate/putamen [8]. BCCAo-induced oligemia has a transient effect on the neocortex and a long-lasting effect on white matter structures [10]. Gradual hippocampal injury coursing with astrogliosis and microglial activation has been observed in animals at several time points after BCCAo. Nonetheless, some of these histopathological changes after BCCAo in rats are strain-dependent [6].

Cerebral blood flow (CBF) after BCCAo shows an acute ischemic phase lasting 2-3 days, followed by a chronic oligemic phase of approximately 3 months, after which the blood flow is restored to normal levels [11,12]. This latter effect is attributed to a key vascular remodeling phenomenon that accompanies long-lasting hypoperfusion. Thus, the occlusion of one carotid artery and both vertebral arteries leads to the enlargement of the ipsilateral posterior cerebral artery 3 weeks after the surgery [11]. Along the same line, Choy et al. [12] demonstrated an increased diameter of various cerebral arteries 6 months after BCCAo, as assessed by post-mortem India ink angiograms. The dilation and enlargement of some of these arteries was confirmed 1 month after BCCAo by magnetic resonance imaging (MRI) angiography [13]. However, the temporal pattern of arteriogenic and parenchymal changes after BCCAo has not been described to date. Here we used a non-invasive quantitative method to assess the progression of morphological alterations of the main cerebral arteries in living rats subjected to BCCAo and carried out longitudinal MRI studies to identify structural and microstructural brain alterations, followed by histological validation of tissue damage. The arterial MRI changes induced by BCCAo were related to CBF and to vascular structural integrity, as assessed in dissected arteries. In addition, several cognitive executive skills were tested longitudinally in these animals using an operant conditioned paradigm, and the results were correlated with the alterations observed by MRI and histology. 

## Materials and Methods

### Animals

Experiments were performed in adult male Wistar rats, weighing 300–320 g at the beginning of the study. Rats were housed in cages under controlled temperature (21 ± 1°C) and humidity (55 ± 10%), with a 12-h light/12-h dark cycle (light between 8:00 AM and 8:00 PM). Food and water were available *ad libitum* during all experiments. Animal work was performed following the local legislation (Decret 214/1997 of July 30th by the ‘Departament d’Agricultura, Ramaderia i Pesca de la Generalitat de Catalunya’) under the approval of the Ethical Committee of the University of Barcelona (CEEA), and in compliance with European legislation.

### Surgery

Rats were anaesthetized under 4% isofluorane in a mixture of 30% O_2_ and 70% N_2_O, and then maintained under 1.5% isofluorane. They were allowed to breath spontaneously throughout surgery. Body temperature was monitored using a rectal probe and maintained between 36.5°C and 37.5°C. Animals were randomly selected for BCCAo (n=17) or sham (n=13) surgery. A ventral midline incision was made to expose the common carotid arteries, which were carefully separated from the vagus nerves. Both common carotid arteries were tied off with two 3-0 sutures (Suturas Aragó, Barcelona, Spain) before the bifurcation of the internal and the external carotids. The first carotid to be occluded, either right or left, was alternated throughout the experiment. Sham animals underwent the same surgical procedure except for the occlusion of the two carotids.

### Magnetic Resonance Imaging

MRI experiments were conducted on a 7.0 T BioSpec 70/30 horizontal animal scanner (Bruker BioSpin, Ettlingen, Germany), equipped with an actively shielded gradient system (400 mT/m, 12 cm inner diameter). The receiver coil was a 4-channel phased-array surface coil for the rat brain. Animals were placed in supine position in a Plexiglas holder with a nose cone for administering anaesthetic gases (1.5% isofluorane in a mixture of 30% O_2_ and 70% CO_2_) and were fixed using a tooth bar, ear bars and adhesive tape. Tripilot scans were used to ensure accurate positioning of the head in the isocenter of the magnet.

For the quantification of the vascular remodeling time-course, time-of-flight (TOF) angiography with a three-dimensional (3D) Fast Low Angle Shot (FLASH) method was acquired before the surgery (pre-occlusion) and 2 h, 24 h, 10 days and 3, 7 and 12 weeks after BCCAo (n=10) or sham-operation (n=6). The scan parameters were as follows: echo time (TE) = 2.5 ms, repetition time (TR) = 15 ms, field of view (FOV) = 35 x 35 x 50 mm, matrix size = 256x 256 x 128 pixels, resulting in a spatial resolution of 0.137 x 0.137 x 0.39 mm.

At the same time points, brain lesions were evaluated longitudinally by T2 mapping of coronal slices acquired with a multislice-multi-echo (MSME) sequence by applying 16 TEs, from 11 to 176 ms, TR = 4764 ms, slice thickness = 1 mm, number of slices = 18, FOV = 40 x 40 mm, and matrix size = 256x 256 pixels, resulting in a spatial resolution of 0.156 x 0.156 mm in 1.00 mm slice thickness.

For registration purposes and longitudinal evaluation of other structural alterations that were not observable in T2 relaxometry maps, high resolution 3D Modified Driven Equilibrium Fourier Transform (MDEFT) images were acquired at the same time points, as above. The scan parameters were as follows: TE = 3.5 ms, TR = 4000 ms, 8 segments and the same geometry as T2 maps except for slice thickness, which was 0.5 mm.

To study microsctructural changes, Diffusion Tensor Imaging (DTI) was performed 7 weeks after BCCAo or sham surgery, using an echo planar imaging DTI sequence with TR = 14500 ms, TE = 30.85 ms, four segments, b-value = 1000, 126 diffusion directions, five B0 images, FOV = 22.23 x 22.23 x 17.92 mm, matrix size = 72x 72 x 58 pixels, resulting in an isometric spatial resolution of 0.309 x 0.309 x 0.309 mm and an acquisition time of 2 h 6 min.

For cerebral perfusion measurements 12 weeks after BCCAo, a T2 * sensitive version of the Echo Planar Imaging (EPI) sequence was used, TE = 18.7 ms, TR = 160 ms, slice thickness = 1 mm, number of slices = 18, FOV = 30 x 30 mm, matrix size = 234x 256 pixels, resulting in a spatial resolution of 0.234 x 0.234 mm in 2.00 mm slice thickness, and 2100 repetitions were recorded consecutively within a total acquisition time of 5 min 36 s. Thus, the recording time per volume was 0.16 s. Gadolinium bolus track experiments were performed in 3 coronal planes. The first 2 min (750 volumes) were used as baseline, then a bolus of 0.5 mmol/kg of gadodiamide (Omniscan™, GE Healthcare Bio-Sciences, S.A., Madrid, Spain) was injected into the femoral vein over a 20-s interval (125 volumes), and the experiment finished with an additional 3 min 36 s of recording (1350 volumes).

A scheme of the timeline for the whole experimental design is shown in Figure S1.

### Image processing

The images resulting from TOF angiography were subjected to a post-processing procedure performed with specific software for scientific image processing and visualization (Amira, Visage Image, Inc). The procedure consisted of several steps, namely signal intensity correction (for the signal decay induced by the surface coil), brain masking, skull stripping, background removal, semi-automatic segmentation of the vertebro-basilar artery, right and left middle cerebral arteries, and azygos-pericallosal artery and, finally, skeletonization of the segmented arteries. The images acquired after BCCAo or sham-operation were affine-registered to their respective pre-occlusion scan so that the same brain mask and artery segmentation could be applied to all time points with minor manual editing. Subsequently, arterial length and arterial tortuosity were measured from the artery skeleton. For vertebro-basilar arteries, the ratio between the hypothetical minimum length and the true length was expressed as the arterial tortuosity. A third parameter related to vascular remodeling was calculated from the Maximal Intensity Projection (MIP) of the cropped base of the brain (Figure S2). The ratio between the area occupied by the projected arteries and the area of the brain projection were calculated for all acquired time points in sham and BCCAo animals.

T2 maps and MDEFT images were reconstructed with Paravision 5.0 software (Bruker Biospin, Etlingen, Germany) and custom-made programs written in Matlab (The MathWorks, Inc., Natick, MA, USA). The volume of focal lesions was estimated in the T2 maps after manual delineation of the region showing increased T2 values (range used: 20-150 ms). The areas were then integrated to calculate the volume. Moreover, the presence and number of hyperintense areas in the striatum and the cortex on MDEFT images were determined for each animal at each acquired time point. All analyses were performed blinded to the treatment and by the same experimenter in order to avoid inter-individual differences.

DTI images were studied using a voxel based analysis (VBA) approach as previously described in [14]. Briefly, all rat brains were registered to a reference brain by means of an affine registration that maximized mutual information of fractional anisotropy (FA) volume followed by an elastic warping based on diffeomorphic demons [15] available in MedINRIA 1.9.4 software. Registered volumes were smoothed with a Gaussian kernel of 3 x 3 x 3 voxels with a standard deviation of one voxel to compensate for possible misregistrations. Voxel-wise statistical test (Mann-Whitney U test) was performed, obtaining the voxels with a statistically significant different distribution of diffusion- related parameters between sham and BCCAo animals. Note that the results may be biased by this choice since VBA requires the definition of a reference brain. In order to avoid such bias and increase the reliability of the results, the VBA procedure was repeated taking all animals as template, and only the regions where differences appeared consistently among the different templates were considered. In this way, the variability produced by the arbitrary choice of the reference template was discarded.

For cerebral perfusion imaging, three regions of interest (ROI) were drawn in each hemisphere from T2*-EPI images (medial prefrontal cortex, caudate-putamen and retrosplenial cortex, Figure S3). The time-course of the signal intensity was calculated for each pixel, and the average values were calculated for each ROI. Data were processed with custom-made programs written in Matlab as previously described [16] with minor modifications. Briefly, in order to obtain relative brain perfusion parameters, we calculated the following: the maximum of the signal intensity curve; the time interval from reaching the gadolinium to the moment of the maximum concentration, or time to peak (TTP); the area under the curve (AUC), which provides an estimation of the relative cerebral blood volume (relCBV); and the estimated mean transit time, calculated as the full width of the curve at half maximum (FWHM). Relative CBF (relCBF) was obtained from the ratio relCBV/FWHM.

### Reversal learning and set-shifting behavioral tasks

A separate group of animals was used for behavioral testing (sham n= 7, BCCAo n=7).

#### Apparatus

Behavioral testing took place within two operant conditioning chambers (30 cm×24 cm×30 cm; Med Associates, Georgia, VT), each placed in a sound-attenuating wooden box fitted with a fan for ventilation and masking of extraneous noise. Each chamber was fitted with two levers located on either side of a centrally positioned food magazine, into which an external pellet dispenser delivering 45-mg pellets was placed (Noyes dustless pellets; Rodent grain-based diet; BioServ, Phymep, Paris, FR). Each chamber had a light-emitting diode (LED) positioned centrally above each lever, a magazine light, and a house light. Magazine entry was detected by an infrared photocell beam located horizontally across the entrance. The apparatus was controlled by MEDPCIV software.

#### Behavioral training and testing

The operant training protocols were based on those described in [17] with minor modifications. Rats underwent a pre-training phase prior to sham or BCCAo surgery to allow familiarization with the testing apparatus. Animals were trained under a fixed ratio of 1 and 3 schedules on each lever separately, followed by the acquisition of a two-lever spatial discrimination task as previously described [18]. Each rat had one training session per day and was trained to reach a criterion of nine correct trials in two consecutive blocks of 10 trials (binomial distribution p < 0.01, likelihood of attaining criterion in a 10-trial block). Once the criterion was reached, the initial discrimination phase was considered complete, and the animal was returned to the home cage. If the criterion was not achieved, this phase was repeated the next day until criterion achievement.

After this training period, animals were randomly separated into groups and subjected to sham or BCCAo surgery. After 10 days of recovery (and acquisition of the second MRI scans), rats were retested on discrimination and once the criterion was achieved the reversal phase started. This process was repeated after each subsequent scan time, namely at 3 and 7 weeks after BCCAo or sham surgery.

#### Set-shifting task

Twelve weeks after BCCAo or sham surgery, animals underwent a final spatial response re-discrimination step, and the set-shifting task was tested as follows. This task required the animal to cease following an egocentric spatial response strategy and instead use a visual-cue discrimination strategy to obtain food reward [19]. The previous random light presented over the levers during the response interval was now the conditioned stimulus, which indicated the active lever. Trials were performed in a manner identical to the initial spatial response discrimination ones. Trials continued until the rat performed nine correct trials in two consecutive blocks of 10 trials. Again, when an animal did not achieve the criterion on the first day of training, it received an identical session of visual-cue discrimination training on the following day.

### Tissue preparation, histochemical and immunohistochemical studies

After the last MRI acquisition (12 weeks), animals were anesthetized with isofluorane and transcardially perfused with saline followed by 4% paraformaldehyde in 0.1M phosphate-buffered saline (PBS) (pH=7.4). Afterwards, the brains were removed and the basilar (BA) and middle cerebral (MCA) arteries were isolated. Brains were postfixed overnight in the same fixative at 4 °C, dehydrated, and embedded in paraffin. The brains were cut (5 µm) in coronal slices at different levels from Bregma (Bregma +3.7, +0.2, and -3.14) following the atlas of Paxinos and Watson (1996) and using a microtome (Leica, Microsystems, Wetzelar, Germany). Cellular damage was assessed by hematoxylin and eosin (HE) staining. Klüver-Barrera Luxol fast blue (Luxol) staining was used to evaluate myelin damage. Immunohistochemistry against glial fibrillary acidic protein (GFAP, a specific marker of astroglial cells) and against Iba-1 (a specific marker of reactive microglia/macrophages) was carried out to reveal signs of glial reaction. The rabbit polyclonal antibodies against GFAP and Iba-1 (Dako, Dakopats, USA) were diluted at 1:500 in PBS containing 0.3% Triton-X100 and 0.2% gelatin. The immunocytochemical procedure has been described elsewhere [20]. Briefly, sections were rehydrated and incubated overnight with the corresponding primary antibody in a humidified chamber at 4 °C and then incubated for 1 h at room temperature with the appropriate biotinylated second antibodies, diluted 1:200 (goat anti-rabbit IgG) (Vector Laboratories, USA). All sections were incubated with avidin-biotin-horseradish peroxidase complex 1:100 (ABC kit, Vectastain, Vector Laboratories) for 1 h. Peroxidase labeling was visualized with a solution containing 0.025% diaminobenzidine (Sigma Aldrich) and 0.03% H_2_O_2_ (Sigma Aldrich) in PBS. In each experiment, a tissue section was also processed in parallel without the primary antibody as a control for non-specific staining.

For quantitative analysis, images of the corpus callosum (CC) and of the optic tract were taken though an optical microscope with a x 40 objective connected to a digital camera, and the analyses were performed using NIH ImageJ software. Myelin fiber density was determined using a threshold that was fixed for all images, and values were expressed as percent density of myelin fibers per field. The number of Iba1-immunopositive cells was counted in fields of 0.075 mm^2^. The quantification was performed in at least 3 representative fields per region and rat, and the numbers were averaged and the values expressed as the mean ± SEM.

The dissected arteries were post-fixed with 4% PFA for 45 min and washed in three changes of PBS. After cleaning, vessels were placed overnight in PBS containing 30% sucrose. They were then transferred to a cryomold (Bayer Química Farmacéutica, Barcelona, Spain) containing Tissue Tek OCT embedding medium (Sakura Finetek, Europe, The Netherlands) for 20 min and frozen in liquid nitrogen. Frozen transverse sections (14µm) of BA and MCA were cut onto gelatin-coated slides and air-dried for at least 90 min. After blockade, sections were incubated with a rabbit polyclonal antibody against collagen I/III (1:30; Calbiochem, Pacific Center Court, San Diego, CA, USA) in PBS containing 2% bovine serum albumin (BSA) for 1 h at 37°C in a humidified chamber. After washing, rings were incubated with the secondary antibody, a donkey anti-rabbit IgG conjugated to CyTM3 (1:200; Jackson Immunoresearch Laboratories Inc., West Grove, PA, USA) for 1 h at 37°C in a humid chamber. After washing, sections were stained with the nuclear dye Hoechst 33342 (0.01 mg/ml; Sigma Aldrich) for 15 min followed by 3 washes in PBS. Immunofluorescent signals were viewed using an inverted Leica TCS SP2 confocal laser scanning microscope with an oil immersion lens (x20). CyTM3-labeled antibody was visualized by excitation at 568 nm and detection at 600-700 nm. The specificity of collagen I/III immunostaining was evaluated by omission of the primary antibody and processed as above. Under these conditions, no staining was observed in the vessel wall in any experimental condition. Quantitative analyses of collagen I/III fluorescence and nuclei number were performed with MetaMorph 4.6 Image Analysis Software (Universal Imaging, Molecular Devices, Downingtown, PA, USA). The intensity of fluorescence per area was calculated in three rings of each animal, and the results were expressed in arbitrary units. To evaluate the number of smooth muscle cells/ring the autofluorescence of the elastic laminae was used to identify the media. In addition, the nuclear shape of the different cell types, visualized by Hoesch 33342, confirms the presence of smooth muscle cells in the media (see Figure S4). The number of nuclei was counted in three rings of each animal, and the results were expressed as cell density. All measurements were conducted in a blind fashion.

### Vessel morphometry

Morphometric assessment of the lumen, media and vessel areas was performed in coronal sections of the dissected arteries using MetaMorph 4.6 Image Analysis Software, as previously described [21]. To determine the luminal area, the cross-sectional area enclosed by the internal elastic lamina was corrected to a circle by applying the form factor l2/4π to the measurement of the internal elastic lamina, where l is the length of the lamina. The vessel area was determined by the cross-sectional area enclosed by the external elastic lamina corrected to a circle by applying the same form factor (l2/4π) to the measurement of the external elastic lamina. Internal and external diameters were calculated from luminal and vessel areas, respectively. Structural parameters were calculated as follows: cross-sectional area (CSA) = le – li; wall thickness (WT) = (De - Di)/2 and wall/lumen (W/L) ratio = WT/Di, where le is the vessel area, li the luminal area, De the external diameter, and Di the internal diameter. Mean values were obtained from three sections of each dissected artery from BCCAo (n=6) and sham (n=5) animals.

### Statistical analysis

The length and tortuosity of the arteries and vascular network quantification were analyzed using a two-way analysis of variance (ANOVA), with treatment (BCCAo- or sham-operated animals) as the ‘between’ factor and time (days) as the ‘within-subject’ factor. When significant overall interactions were found, further analyses of partial interactions were carried out. Post-hoc analyses for intergroup comparisons were performed with Fisher’s least significant differences (LSD) test when the initial p value was significant. All data were analyzed using Statistica software (StatSoft Inc., France). Differences were considered significant when *P*< 0.05. All results are expressed as mean ± SEM. Data from arteries and brain histology and immunohistochemistry, and gadolinium bolus track measurements and behavior were analyzed using an unpaired Student’s t-test and two-way ANOVA, respectively. In all cases, data analysis was carried out using GraphPad Prism 4 Software. A value of *P*< 0.05 was considered significant.

## Results

### Tissue alterations assessed with MRI

**Figure 1:**
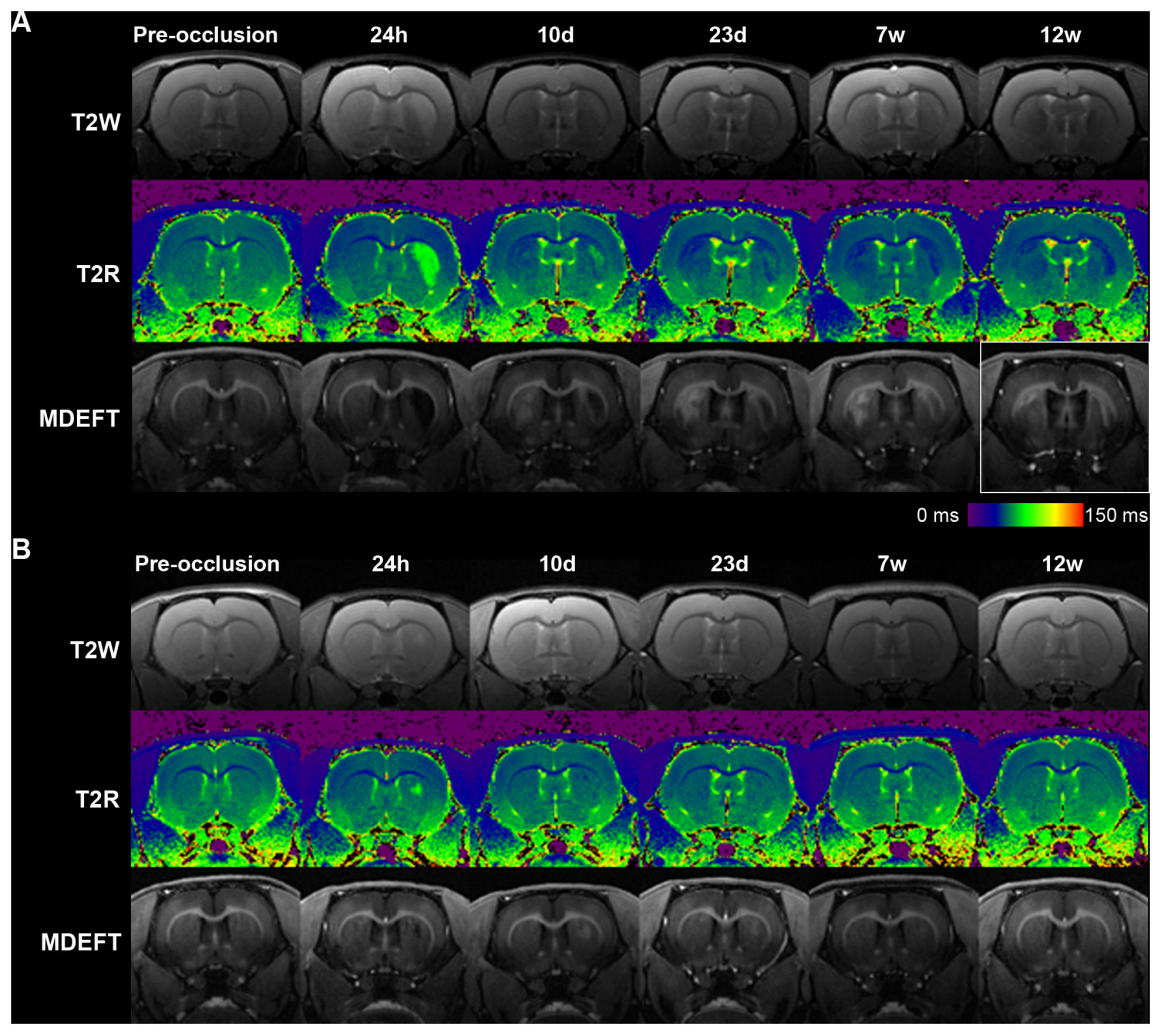
Longitudinal multimodal MRI. Representative coronal section of two BCCAO animals (A, B) are shown at different time points. BCCAO induced variable degrees of focal acute ischemic lesions (see right striatum at 24 h in A and B) and delayed damage (see left striatum from day 10). For both animals (A, B), images in first row are T2-weighted (T2W), second row are T2 relaxometry map (T2R) and third row are 3D MDEFT T1-weighted (MDEFT) images. The box highlights MDEFT alterations at 12 weeks in the same animal shown in Figure 3 for histological damage. Color scale represents T2 relaxometry time (ms) for images shown in second row of panels A and S (T2R).

The presence of acute ischemic focuses was evaluated with T2 relaxometry mapping 24 h after BCCAo or sham-operation. In 70% of the rats, BCCAo induced unilateral focal acute damage manifested as areas of increased T2 values located in the striatum with widely variable sizes ranging from 1.7 mm^3^ to 27.13 mm^3^. Acute lesions tended to be more frequent in the right (6 out of 10) than in the left (3 out of 10) striatum, but differences were not statistically significant (Chi-square test = 1.82, *P*> 0.05). Figure S5 shows an incidence map of all ischemic lesions observed 24 h after BCCAo. The same lesions were observed as hypointense zones in the 3D MDEFT T1-weighted images. Coronal sections of two representative BCCAo animals illustrating the largest and smallest tissue lesion are shown in Figure 1A and 1B, respectively, at several time points and with the MRI modalities used in this study. In the MDEFT images, acute damage was observed as a hypointense zone while adjacent areas and secondary injuries were seen as hyperintense zones (Figure 1). The size of the acute MDEFT hypointense areas decreased with time and was progressively substituted by hyperintense signals that surrounded the hypointense core. MDEFT provided a contrast superior than that obtained in T2-weighted images and maps for the progressive evolution of the tissue lesion.

Bilateral striatal increases in T2 values were not observed 24 h after BCCAo; however, in 20% of the rats, bilateral alterations became apparent at day 10. These delayed MRI alterations were manifested as hyperintensities in MDEFT from day 10 onwards, corresponding with decreased T2 values in regions showing no apparent T2 alterations at 24 h (see left striatum at 24 h in upper and middle row of Figure 1A). These lesions were therefore more delayed than the acute ischemic lesions. In addition, they showed distinct MRI features to those observed in the acute ischemic zones and were found in subcortical regions (striatum).

**Figure 2:**
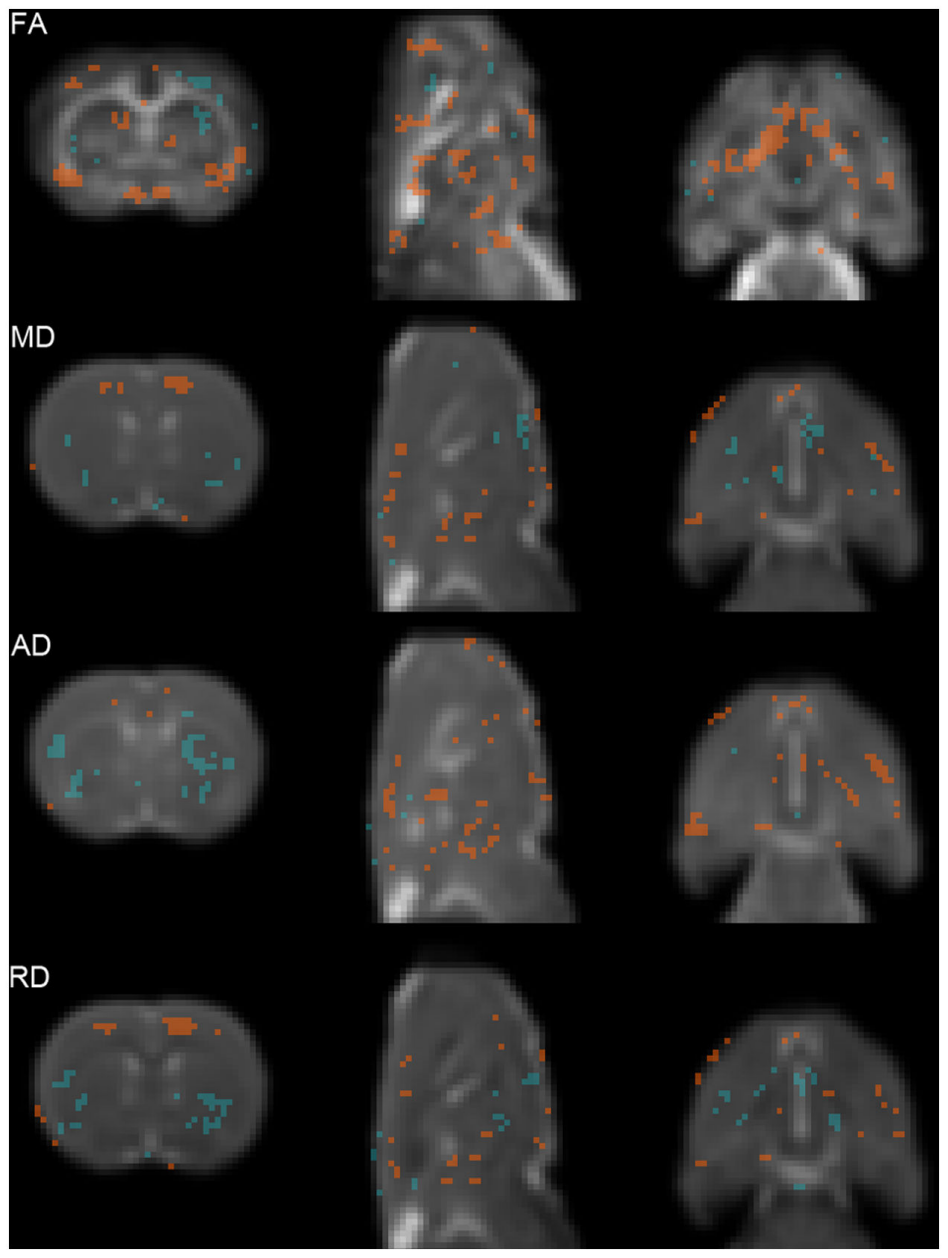
Statistical maps showing Diffusion Tensor Imaging (DTI) indexes. Regions showing statistically significant differences of fractional anisotropy (FA), mean diffusivity (MD), axial diffusivity (AD) and radial diffusivity (RD), between sham and BCCAo animals 7 weeks after surgery. Background images are the corresponding averaged DTI index calculated for the sham group (left: coronal, middle: sagittal, and right: axial slices). Red areas (sham > BCCAo) have a significance of *P* <0.05 (Mann-Whitney U test). Blue areas (sham < BCCAo) have a significance of *P* <0.05 (Mann-Whitney U test). The slices show representative anatomical structures (see Results section for details).

DTI identified alterations in multiple grey and white matter areas (Figure 2). Indeed, VBA showed a significant decrease in FA values in BCCAo compared to sham animals in structures such as the piriform, frontal and insular cortical regions, hippocampus, thalamus, and optical nerve and tract, while significant increase in FA values was observed in the lateral striatum (Figure 2, FA). Mean and radial diffusivities (Figure 2, MD and RD) were significantly reduced in the cingulum and increased in the lateral striatum of BCCAo versus sham rats. Furthermore, axial diffusivity (AD) was significantly increased in the striatum of BCCAo compared to sham rats, possibly as a result of the multiple focal lesions previously described in some animals (Figure 2, AD). The means of the four DTI indexes for the above-described areas are shown in Table S3.

### Immunohistochemical evaluation of brain tissue alterations

#### Relation of MRI changes and histological damage

**Figure 3:**
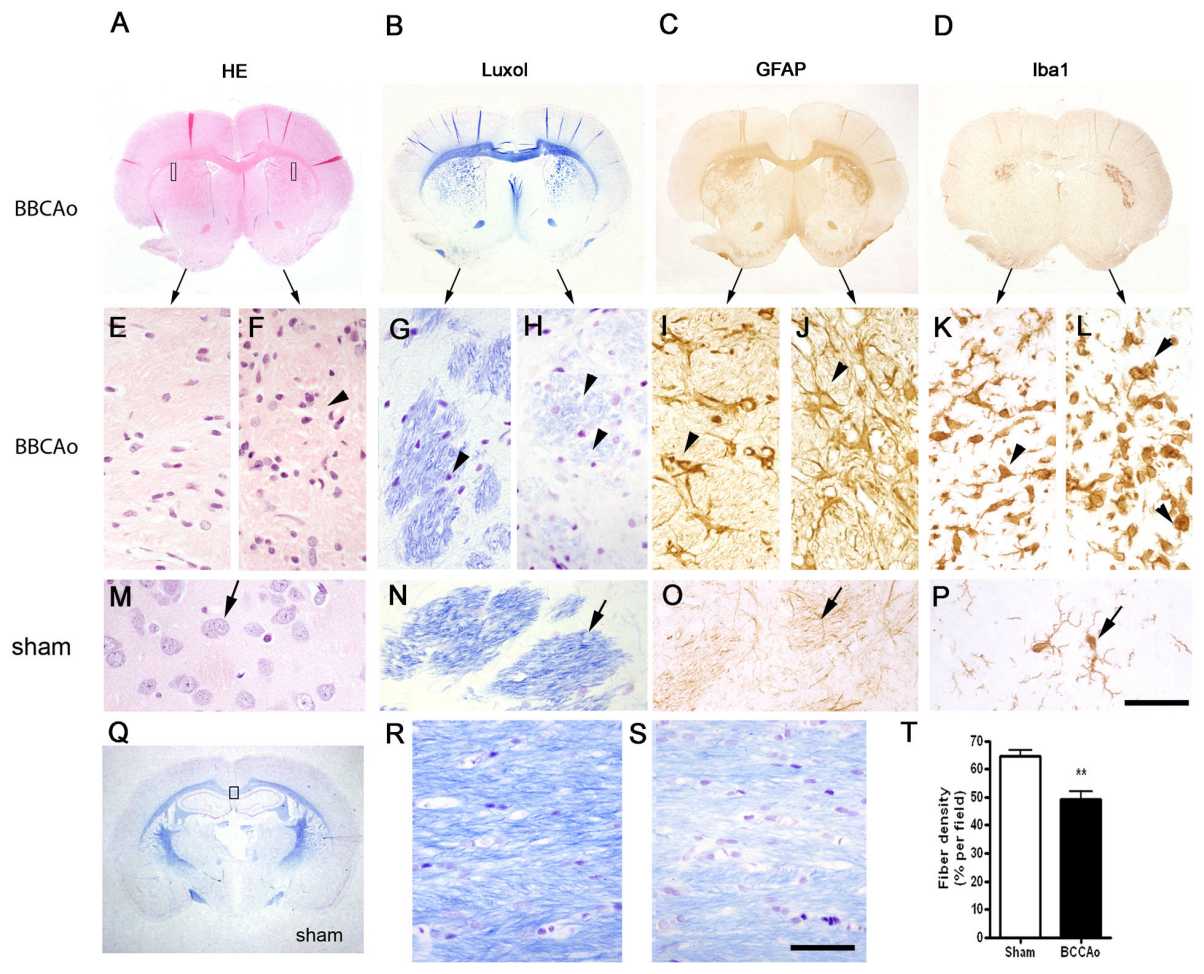
Histological evaluation of brain lesions 12 weeks after surgery. A-D) Macroscopic images of coronal brain sections (at the level of Bregma +0.2) of a BCCAo rat showing bilateral alterations in the striatum. This animal corresponds to the same rat shown in the MRI image in Figure 1A. Sections show tissue structure (hematoxylin-eosin, HE) (A), myelinated fiber tracts (B), and astroglial (GFAP) (C) and microglial (Iba1) (D) reactions. Corresponding magnifications of the above images are shown in the middle row (C-L) for the areas illustrated with rectangles in the image (A). Alterations (arrowheads) are apparent in both hemispheres compared to the tissue of a sham-operated rat (M-P). Arrows in M-P point to healthy structures and cells. The features of the alterations in the left and right hemisphere of this BCCAo rat differ, with more prominent changes in the right striatum, thus illustrating the variability in the extent and severity of the damage after BCCAo. Q) Luxol staining of a brain section (Bregma -2.3) indicating with a rectangle the genus region of the corpus callosum shown in higher magnification in (R) and (S) for a sham-operated and BCCAo rat, respectively. BCCAo induces rarefaction with pallor of the fiber staining and vacuolization (arrowheads). T) Quantification of fiber density shows a significant reduction in the genus of the corpus callosum 12 weeks after BCCAo. Data are represented as mean ± SEM. ** *P*< 0.01 (Unpaired Student’s t-test). Scale bar a-d, q = 0.5 cm; e-p, r, s = 50 µm.

Necrotic zones (hematoxylin-eosin) with activated microglia/macrophages (Iba-1+) surrounded by an astroglial scar (GFAP+) (Figure 3A-D) were observed at 12 weeks in the striatum of all BCCAo rats previously showing an acute increase of T2 values (hypointensities in MDEFT images). Histological alterations were not detected in sham-operated rats. The anatomical distribution of the areas showing glial reaction matched those affected in MRI. In an attempt to relate hyper- and hypo-intensities in MRI images with the type of histological changes, we show the same rat in Figure 1A and Figure 3. The zone that was hypointense in the right striatum in MDEFT (Figure 1A) at 12 weeks (time of postmortem histological study) corresponds to a necrotic core that was surrounded by a strong glial reaction (Figure 3C-D, I-L) manifested as a hyperintense ring in MDEFT images (decreased T2 values in relaxometry maps) (e.g. right striatum in Figure 1A highlighted in the box). In addition to the acute MRI alterations, delayed MRI changes manifested as hyperintense signals in 3D MDEFT T1 images (e.g. left striatum in Figure 1A in the section highlighted in the box) (decreased T2 values in relaxometry maps) in the absence of acute T2 alterations were seen in some animals. These MRI changes corresponded to zones of abundant gliosis at 12 weeks. The presence of gliosis in zones lacking signs of acute infarction is compatible with progressive neuronal death and/or axonal degeneration subsequent to insufficient blood supply after BCCAo.

#### White matter alterations

Alterations in the white matter, as assessed with Luxol Fast blue myelin staining, were manifested by the disarrangement of the nerve fibers, the formation of marked vacuoles, and the disappearance of myelinated fibers (Figure 3G, H, S). These pathological changes were accompanied by astrogliosis (Figure 3I, J) and microgliosis (Figure 3K, L) and were seen in all striatal regions showing acute MRI changes (e.g. Figure 3H, J, L vs. Figure 1A-right striatum), in agreement with the DTI changes observed at the same level (Figure 2, decrease in FA and increased AD in the striatum). To a lower extent, astrogliosis and microgliosis were also detected in regions that did not show acute MRI alterations (Figure 3G, I, K vs. Figure 1A-left striatum). Regions showing severe white matter alterations included the optic tract (manifested in all BCCAo rats, see below), the fiber bundle of the caudate-putamen (60% of BCCAo rats), and –with less intensity- the CC. Densitometric analysis in the genus of the CC showed a statistically significant reduction in myelin fiber density (*P*< 0.01) (Figure 3t). These histological results are in agreement with the decreased FA observed in the optic tract and nerve (Figure 2).

#### Optic tract degeneration

**Figure 4:**
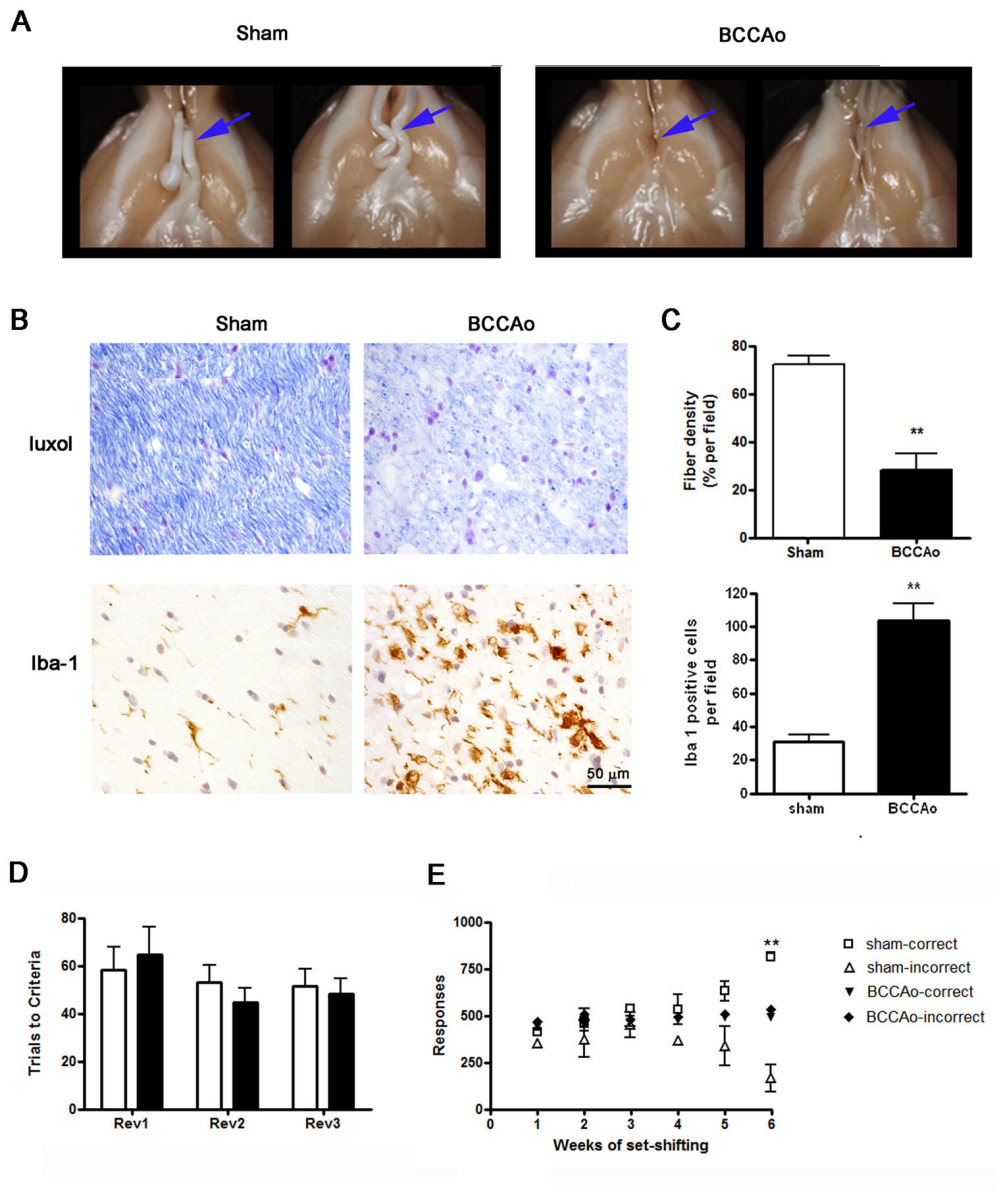
Signs of optic tract degeneration 12 weeks after surgery. A) Macroscopic images of the optic tract (arrows) show reduce fiber volume in BCCAo rats compared to sham-operated rats. B) Histological evaluation of fiber density assed with Luxol staining of the optic tract (at the level of Bregma -2.3) and immunostaining to show microglia reactivity (Iba-1). C) Quantification of fiber tract density and number of Iba-1+ cells per field (objective x40) show significant increases in BCCAo rats (n=5) versus sham rats (n=3). Data are represented as mean ± SEM. ** *P*< 0.01 (Unpaired Student’s t-test). D) Reversal learning executive function as expressed by trials performed correctly to acquire each reversal test, white and black bars represent sham (n=4) and BCCAo animals (n=7) respectively. C) Set-shifting executive function as expressed by correct and incorrect responses during the 6 testing weeks in sham (n=4) and BCCAo animals (n=7). Asterisks indicate significant differences between correct and incorrect responses (Bonferroni test). ** *P*< 0.01.

Twelve weeks after surgery, all BCCAo rats showed macroscopic signs of optic nerve damage with varying degrees of degeneration affecting the total or partial length of the tract. Figure 4A shows macroscopic images of representative perfused brains (ventral view) of sham-operated rats showing preserved optic tracts and of BCCAo rats showing complete and partial degeneration of the optic nerves before and after the optic chiasm. At the microscopic level, the optic tract of all animals that underwent BCCAo showed reduced fiber density and vacuolization (Figure 4B), astrogliosis (not shown), and microgliosis (Figure 4B). The quantitative analyses showed a statistically significant reduction in fiber density and increased number of microglia cells after BCCAo (*P*< 0.01) (Figure 4C).

#### Grey matter alterations

In the hippocampus, eosinophilic neurons were detected in the CA1 pyramidal cell layer and dentate gyrus. In addition, groups of eosinophilic cells were observed in discrete areas of the cerebral cortex after BCCAo. These neuronal changes were not accompanied by manifested glial reactions (data not shown).

### Behavioral alterations

Both sham and BCCAo groups performed the reversal learning task in a similar manner. There was no significant difference in the ability of the two groups to remember the previously learned discrimination at any time point (data not shown). Nor was there a difference in the number of trials required to reach the reversal learning acquisition criterion at any time tested (F_2,24_ = 0.6718; ns) (Figure 4D). However, regarding the shift of animals from an egocentric spatial response strategy to a visual-cue discrimination strategy to obtain food reward (set-shifting task), the BCCAo rats were unable to achieve the acquisition criterion (Figure 4C). Two-way ANOVA showed time effect: F_5,40_ = 6.40; *P*< 0.001, surgery effect: F_3,40_ = 40.75; *P*< 0.01, and a significant interaction (F_5,40_ = 33.87; *P*< 0.001). Indeed, after 4 weeks of set-shifting training sessions, sham animals started to give more correct than incorrect responses, and the number of correct responses was significantly different after 6 weeks (*P*< 0.01). Significant differences between groups were also observed 6 weeks after the beginning of the set-shifting training (*P*< 0.01).

### MRI longitudinal quantification of arteriogenesis after BCCAo

**Figure 5:**
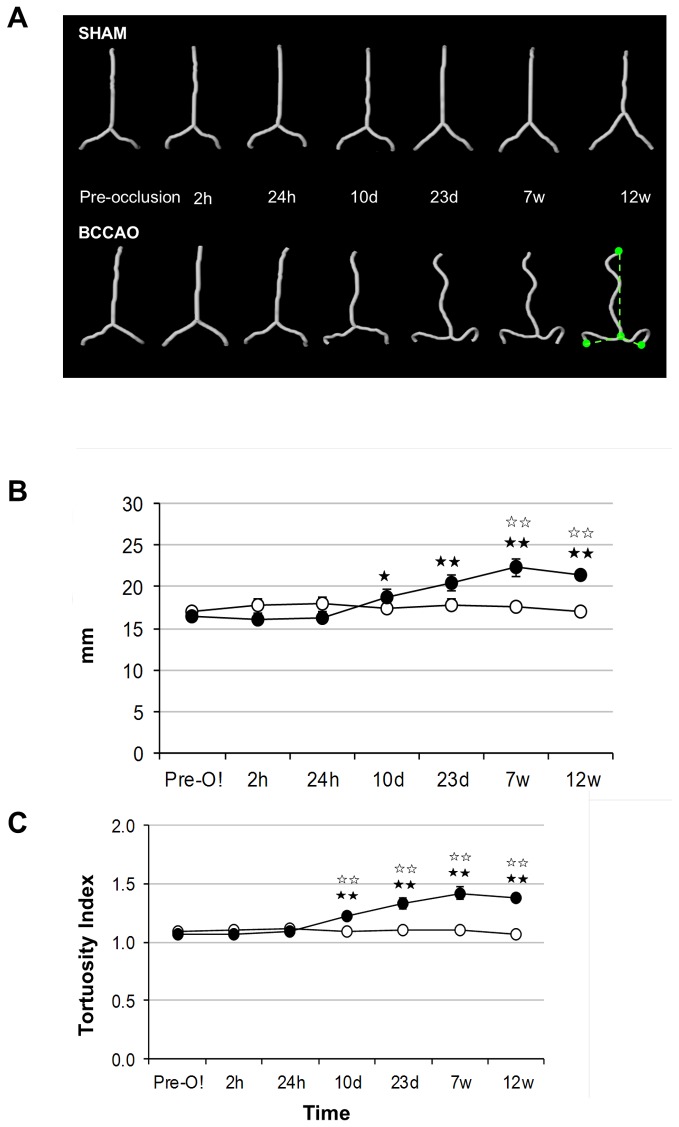
Time course changes in vertebro-basilar artery length and tortuosity following BCCAo in rats. A) Segmentation of the vertebro-basilar artery for representative sham and BCCAo rats for all the time points evaluated. The ratio between the hypothetical minimum length and the true length was expressed as the arterial tortuosity (bottom right picture) (B, C) Measurements of length and tortuosity of the vertebro-basilar arteries (see Methods). The minimal distance from the base of the basilar artery to the final points evaluated is schematically drawn (in green) on the bottom right image of panel a. Vertebro-basilar artery length (B) and tortuosity (C) progressively increased after BCCAo from day 10. A measure of the effect size was determined by calculating the eta squared (η2), which for the treatment effect over the length was 0.077 and 0.242 over the tortuosity, suggesting 7% of the effect observed in length and 24% of the effect observed in the tortuosity accounts for BCCA occlusion. White and black circles represent mean±SEM for sham (n=4) and BCCAo (n=6) groups, respectively. * *P*< 0.05, ** *P*< 0.01.

The temporal pattern of arterial changes after BCCAo was quantified in the main craniocerebral arteries: 1) the vertebrobasilar system, including both vertebral arteries, from its insertion at the dura mater, until the most anterior end of the basilar artery, right at its bifurcation with the posterior cerebral arteries; 2) the left and right middle cerebral arteries; and 3) the azygos-pericallosal artery, which corresponds to the intracerebral segment of the unified anterior cerebral arteries [22]. Notably, the length and tortuosity of the vertebrobasilar system were strongly altered after BCCAo (Fig. 5A). The results of the statistical analyses (two-way ANOVA) are shown in Table S1 for each artery. Significant effects of surgery (BCCAo vs. sham-operation or vs. pre-occlusion data from the same rats) were observed for both the length and tortuosity of the vertebrobasilar artery. Compared to pre-occlusion data, a significant increase in the vertebrobasilar length was found from day 23 after BCCAo (*P*< 0.01) (Fig. 5B). Compared to sham-operated rats, significant differences were also found at 7 and 12 weeks after BCCAo (*P*< 0.01). Similar results were observed for the increase in vertebrobasilar artery tortuosity (Fig. 5C). However, BCCAo did not affect the length or tortuosity of the middle cerebral and the azygos-pericallosal arteries (Figure S6). These results support the notion that the obstruction of blood flow in the carotid arteries promotes compensatory adaptive changes in the vertebrobasilar system, in agreement with previous reports [6,7]. Using MRI arteriography, here we demonstrate that arterial changes were already present one week after BCCAo, they persisted for at least 12 weeks, and they were most abundant in the vertebrobasilar tree. Significant differences were not detected in arterial branches of the internal carotid tree, although we cannot exclude small changes.

**Figure 6:**
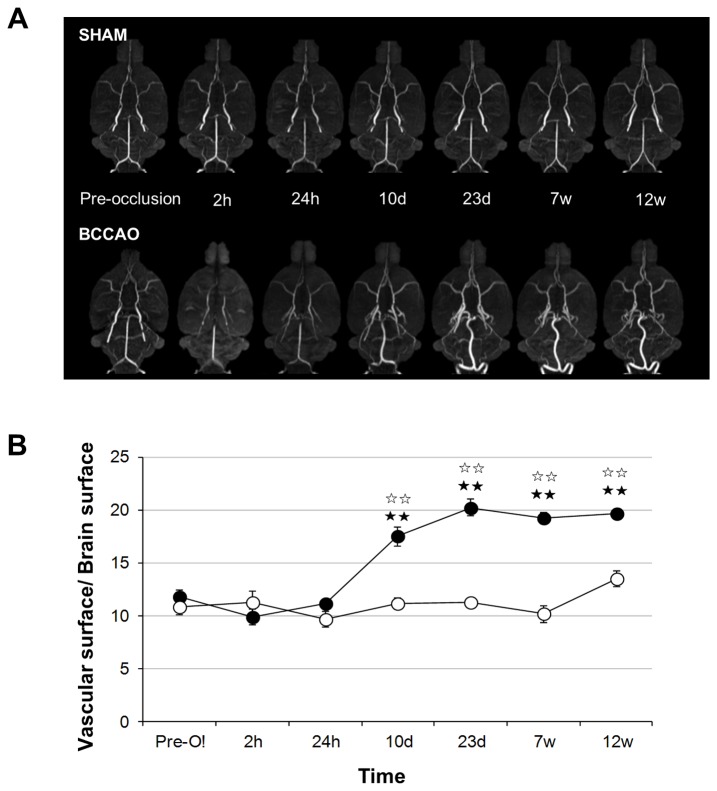
Total arterial area progressively increases after BCCAo. A) Maximal intensity projection (MIP) of the brain basal segment and arteries from representative sham (n = 4) and BCCAO (n = 6) rats at all the time points evaluated. B) Ratio between the brain surface and the vascular surface of the previous MIP calculated at all the time points evaluated. Data are represented as mean ± SEM. Black stars indicate significant differences versus the pre-occlusion data (Fisher’s LSD test), and white stars significant differences versus the sham-operated animals at corresponding time points. ★ P< 0.05; ★★ P< 0.01.

In addition, after BCCAo, the presence of new arteries was detected, especially at the base of the brain (Figure 6A). The temporal characterization of the arteriovascular area revealed a significant effect of BCCAo versus sham-operation (or versus pre-occlusion data) that was time-dependent two-way ANOVA, time effect: F_5,40_ = 16.160; *P*< 0.001, surgery effect: F_1,8_ = 32.749; *P*< 0.001, and a significant interaction (F_5,40_ = 16.295; *P*< 0.001). Subsequent post-hoc analyses revealed significant differences between the two experimental groups at all time points (*P*< 0.01 for all cases), and between pre-occlusion data and certain time points (10 days, 23 days, 7 weeks and 12 weeks after BCCAo) (Figure 6B).

### Structural alterations of the basilar artery 12 weeks after BCCAo

**Figure 7:**
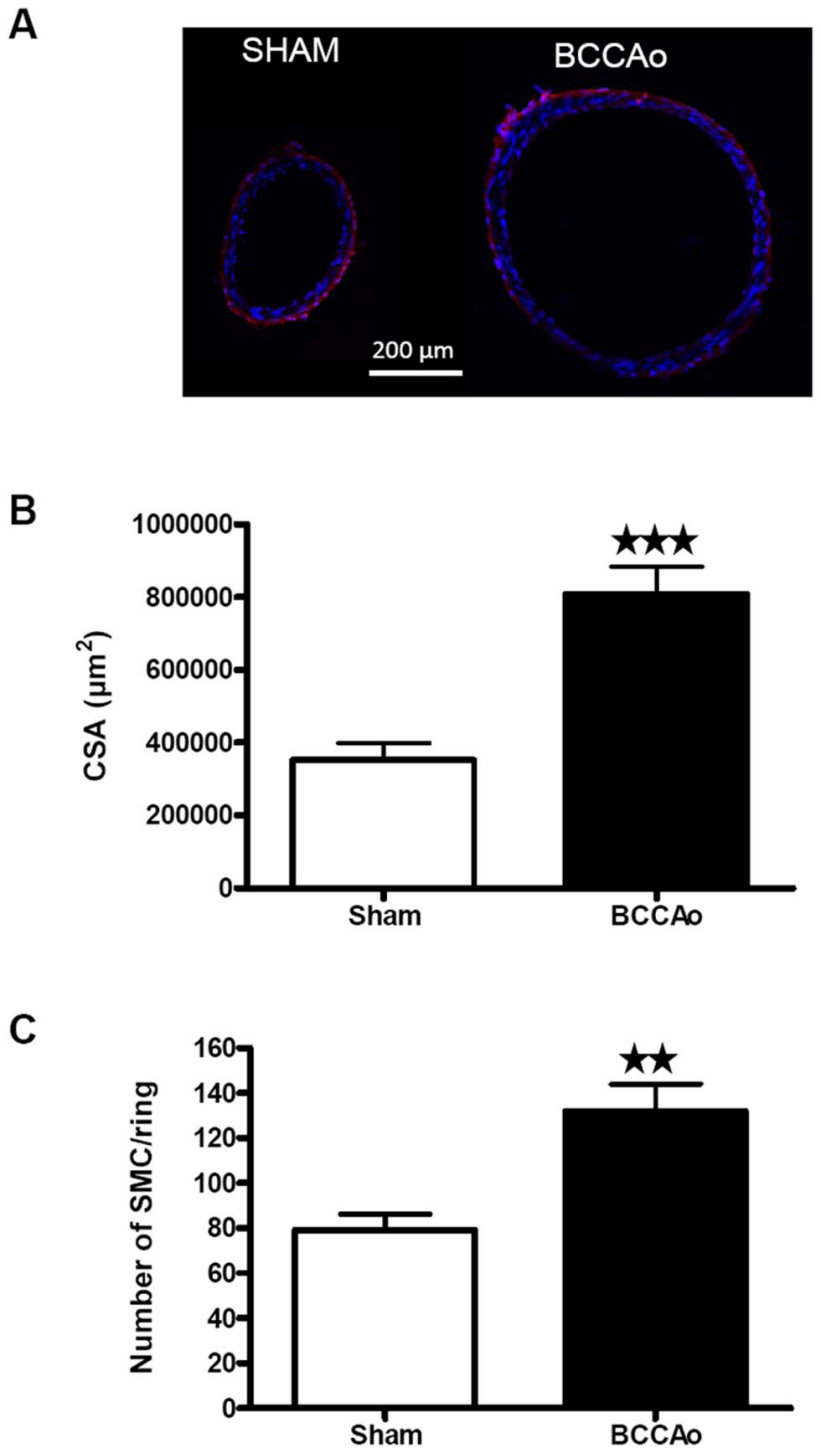
Structural evaluation of the basilar artery (BA), 12 weeks after BCCAo. A) Representative coronal sections of the BA from a sham and a BCCAo rat. Red shows collagen immunostaining and blue shows the cell nuclei. B) Cross-sectional area and C) total number of smooth muscle cells (SMC)/ring calculated for the BA of sham (n = 5) and BCCAo (n = 6) group 12 weeks after surgery. Data are represented as mean ± SEM. Asterisks indicate significant differences between the 2 groups. ★★ *P*< 0.01, ★★★ *P*< 0.001 (Unpaired Student’s t-test).

The post-mortem morphometric study of the basilar artery (BA) 12 weeks after surgery showed a significant increase (*P*< 0.001) in vessel (sham: 1147.25 ± 90.42 µm, n=5; BCCAo: 2177.8 ± 102, n=6) and lumen (sham: 942.16 ± 78.78, n=5; BCCAo: 1929.9 ± 92.5 n=6) diameters, which lead to a greater (*P*< 0.001) cross-sectional area (CSA; Figure 7B) and a smaller (*P*< 0.05) wall/lumen ratio (sham: 95.9±5.01 µm, n=5; BCCAo: 123.98±6.8 µm; n=6). These changes were paralleled by an increase (*P*< 0.001) in the total number of smooth muscle cells per ring (Figure 7C). This observation suggests smooth muscle cell proliferation and is compatible with circumferential wall stress caused by artery occlusion [23]. Moreover, these changes were accompanied by a decrease (*P*< 0.05) in collagen I/III fluorescence intensity/µm^2^ in BCCAo rats (35.1 ± 6.3, n=6 vs. 97.48 ± 16.9, n=5 in sham-operated respectively). Taken together, these findings are compatible with outward hypertrophic remodeling [24] of the BA after BCCAo. However, differences in the MCA were not statistically significant (see Figure S7).

### Dynamic susceptibility contrast imaging for evaluation of cerebral perfusion

**Figure 8:**
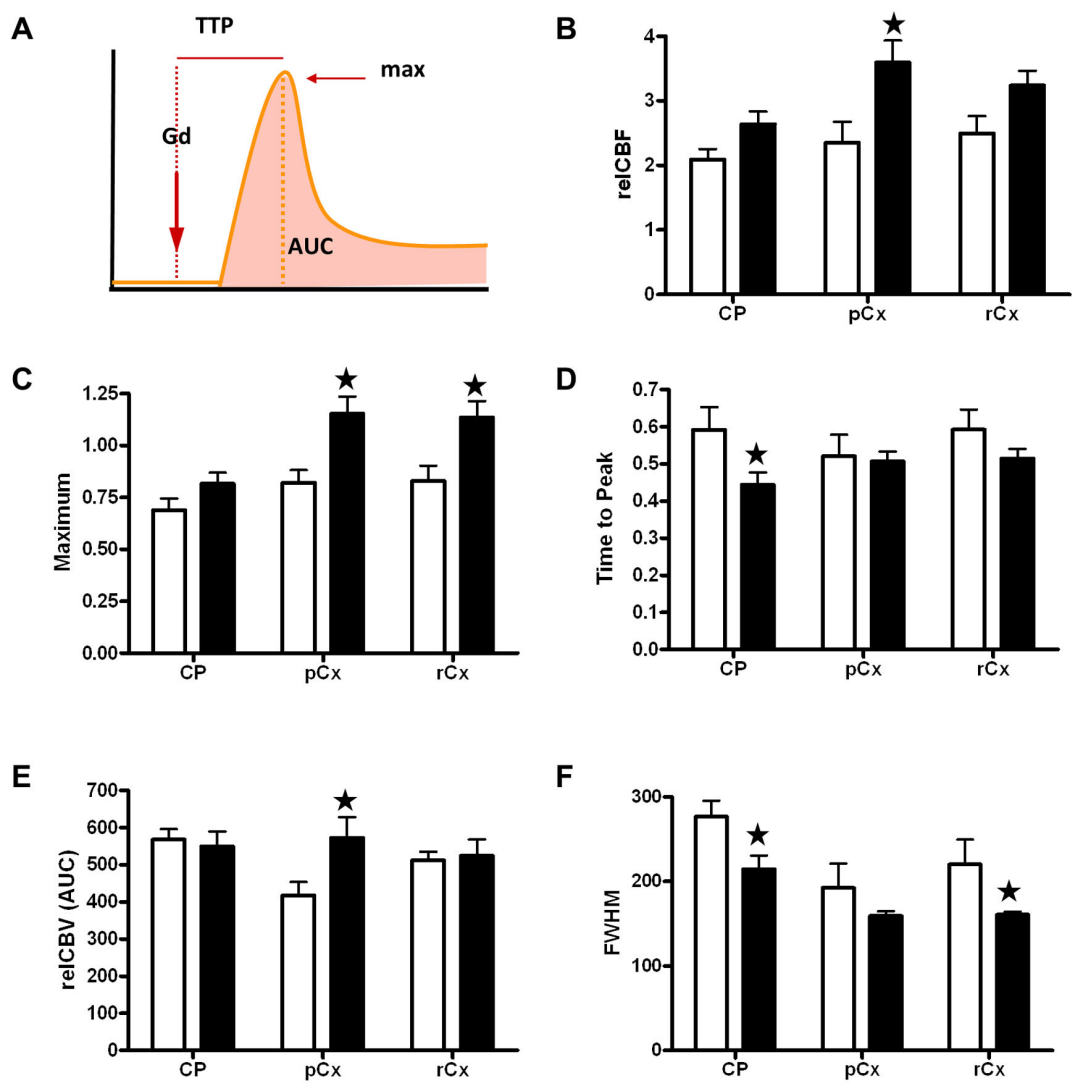
Dynamic susceptibility contrast imaging for evaluation of cerebral perfusion. A) Scheme of the gadodiamide (Gd) bolus track and the evaluated perfusion-related parameters. The maximum (max) is the peak of signal intensity. Time to peak (TTP) is the period of time from gadodiamide injection to the peak of signal intensity. The area under the curve (AUC) provides an estimation of relative cerebral blood volume (relCBV). B) Relative cerebral blood flow (relCBF); C) maximum of signal intensity; D) time to peak; E) relative CBV (relCBV); F) full width at high maximum (FWHM) as an estimation of the mean transit time in several brain regions. White bars represent sham animals (n = 3) and black bars BCCAO animals (n = 7), 12 weeks after surgery. Black stars indicate significant differences between groups (Bonferroni test). ★ *P*< 0.05 CP: caudate-putamen; pCx: medial prefrontal cortex; rCx: retrosplenial cortex.

Measures of relative CBF (relCBF) (Figure 8B) that were carried out by dynamic susceptibility contrast imaging demonstrated that cerebral hypoperfusion was not present 12 weeks after BCCAo. On the contrary, a subtle cerebral hyperperfusion was apparent at this time in the BCCAo group, since two-way ANOVA by type of surgery and brain region showed a significant effect of surgery (F_54,1_=14.97; *P*< 0.01), whereas no regional effect (F_54,2_=5.91; n.s) or interaction between these two factors (F_54,2_=1.82; n.s) was observed (Figure 8B). The statistical analysis for maximum gadolinium-induced signal enhancement, TTP, relCBV (AUC) and FWHM are shown in Table S2. Briefly, significant effects of BCCAo were demonstrated in various structures for maximum gadolinium-induced signal enhancement, TTP and FWHM (respectively Figure 8C, D and F). Taken together, our results show that the hypoperfusion induced by BCCAo was transient since it was no longer observed 12 weeks after surgery. 

## Discussion

Here we describe the dynamics of brain tissue alterations and the phenomenon of arteriogenesis arising after BCCAo in rats, as assessed non-invasively by longitudinal multimodal MRI. The MRI results were found to be related to specific histopathological and behavioral alterations. BCCAo induced variable degrees of acute ischemia in brain tissue, with some animals showing clear signs of acute ischemic damage that were absent in other animals. Furthermore, damage ranged from small subcortical lesions, which might have some features resembling lacunar infarctions, to extensive infarctions involving most of the striatum. This variability might not be merely a feature of the experimental animal model since, to some extent, it is reminiscent of that seen in the clinical evolution of patients with internal carotid artery occlusion. In these cases, some individuals remain asymptomatic while others suffer recurrent transient ischemic attacks (TIA) or various types of stroke [25]. BCCAo in rodents has been proposed as an experimental model of vascular dementia and small vessel disease [26,27,28] induced by progressive brain damage. However, the variability in acute tissue damage precludes its use for studying these diseases unless animals were selected in the acute/sub-acute phase with predefined MRI criteria, i.e. including animals with very small subcortical lesions or with no detectable acute lesions, but excluding those with large acute infarctions. Acute brain lesions in this experimental model are attributable to the sharp drop in CBF caused by sudden BCCAo [12,29]. Novel implementations to the BCCAo model through the application of constrictor devices that induce a progressive rather than a sharp drop in CBF might be advantageous [29], providing that the degree and dynamics of hypoperfusion can be standardized in the animals.

Acute ischemic lesions after BCCAo tended to be more frequent in the right than in the left striatum. This effect might be attributable to interhemispheric differences in vascular anatomy and/or neuroanatomical and neurochemical properties. Several experimental evidences support the latter possibility. For instance, more GABA binding sites have been reported in the left than in the right striatum [30]. Likewise, dopamine D2 receptors are more abundant in the left compared to the right striatum in adult male rats [31]. In a model of transient forebrain ischemia (4-vessel occlusion) in rats, the inhibitory synaptic transmission was stronger in the left than in the right striatum [32]. Therefore, it is possible that the left striatum has richer GABAergic activity than the right striatum in the rat. This and other functional asymmetries might underlie a higher neuronal vulnerability to ischemia in the right striatum and might contribute to explain the tendency for a higher incidence of acute infarction in the right than in the left striatum after BCCAo in rats.

Acute brain lesions after BCCAo must be distinguished from the alterations caused by long-lasting hypoperfusion, which is associated with changes in the white matter that evolve with blood vessel rarefaction, axonal loss, myelin vacuolization and demyelinization [33], with damage to myelin preceding that to the axon [34]. More recent data disclosed the rapid effect of hypoperfusion (3 days), disrupting proteins involved in the stability of axon-glial connection and causing damage first to the paranodal septate-like junctions and later to the septate-like junctions [35]. White matter alterations are preceded by changes in blood vessel structure and blood-brain barrier (BBB) permeability [36]. The latter variations approximate BBB alterations described in aged humans that might contribute to small vessel disease [37,38]. The small white matter content in rodents, together with the small size of the brain, precludes an imaging parallelism in animals for leukoaraiosis. Nonetheless, white matter lesions in this experimental animal model have some histological features that may, to some extent, correspond to the radiological signs of leukoaraiosis in humans [2]. Also, chronic BBB alterations with accumulation of toxic components of amyloid precursor protein have been described in the perivascular space of the periventricular white matter of rats after cardiac arrest [39]. Indeed, we have demonstrated that DTI has is able to either show the signs of acute focal lesions (i.e. increase of AD in striatum, Figure 2) which might reflect the glial scar formation previously reported by others [40]. Moreover, DTI reflected diffused white and grey matter damage (i.e. decrease FA in the optic tract, decreased MD and RD in the cingulum, Figure 2). Our results strengthen the hypothesis that diffusion weighted imaging techniques are promising tools to improve the diagnostic of neurodegenerative diseases that present diffuse neuronal damage. In this regard, DTI is a highly sensitive marker of white matter damage in small vessel disease and has been proposed as a potential outcome measure (or biomarker) in clinical trials for drugs designed to treat vascular cognitive impairment (VCI) [41]. Thus, future experimental studies in animal models of vascular dementia by means of DTI and cognitive function correlation will be of great relevance. In addition, in our study, delayed imaging alterations related to gliosis were identified by MRI using the MDEFT sequence. Therefore, this sequence might be useful to image brain alterations in chronic experimental models of neurodegenerative diseases that are accompanied by gliosis.

One discriminatory symptom between Alzheimer’s and VCI patients is impaired executive function since both diseases show memory deficits. In this regard, rodents with partial [42] or complete BCCAo [29,43] show impaired spatial working memory. Here we addressed reversal learning and set-shifting, two components of executive function [19], in order to assess whether BCCAo impaired this function in rats. These behavioral animal studies required tasks involving visual recognition. However, we show that BCCAo in rats causes optic nerve degeneration and visual dysfunction, in agreement with previous observations [44,45,46]. The main arterial blood supply to the optic nerve in the rat comes from the ophthalmic artery, which then trifurcates into the central retinal artery and two posterior ciliary arteries [47]. The ophthalmic artery is a branch of the palatine artery derived from the pterygopalatine artery, which is in turn a large branch of the internal carotid artery [48]. Therefore, BCCAo in the rat causes a strong impairment of blood supply to these branches of the internal carotid artery, causing consistent optic nerve degeneration. In agreement with these findings, visual alterations have also been reported in patients after carotid artery occlusion [49]. The eyes of Alzheimer’s disease patients with related changes in the retina show degeneration and loss of retinal neurons, a reduction of retinal nerve fibers, an increase in optic disc cupping, retinal vascular tortuosity and thinning, and visual functional impairment [50]. All together, these observations suggest that optic nerve and retinal degeneration following vascular pathology might be useful to study the mechanisms underlying neuronal degeneration associated with dementia. Although visual dysfunction may be part of the neurodegenerative disorder, assessing cognitive function in animals with tests involving visually cued tasks may not be fully conclusive since visual alterations in the absence of memory or executive function alterations cannot be excluded. Indeed, our results show that that animals subjected to BCCAo “remember” the position of the levers in the box and preserve the capacity to properly perform the spatial-response tasks. However, BCCAo rats showed worst performance than the sham-operated group when visual cues were required to perform the executive function learning task. This effect was related to the observed optic nerve degeneration, which impaired visual function. Importantly, this result questions the validity of some behavioral studies using this experimental model [51,52,53], since BCCAo rats may not correctly perform visually cued tasks, such as the Morris water maze. To overcome this technical issue, Kalesnykas and colleagues [54] proposed the use of non-visual learning paradigms to test memory dysfunction after BCCAo in rodents.

BCCAo also triggered a progressive process of vascular remodeling, causing potent arteriogenesis in the vertebrobasilar tree that was already detected at day 7 and increased until the end of the study (12 weeks). Here we used a new non-invasive quantitative method to assess the progression of morphological alterations of the main cerebral arteries in living animals. These vascular alterations in arteries that are not directly affected by the occlusion could be attributable to the recovery of blood flow that we observed 12 weeks after BCCAo and that may take place even earlier [55]. Recovery from the induced hypoperfusion is compatible with compensatory adaptive changes triggered by arteriogenesis, in agreement with previous findings [6,7,13]. Structural changes in the basilar artery included an increase in the cross-sectional area, and they are globally compatible with outward hypertrophic remodeling, which is caused mainly by smooth muscle hyperplasia. In contrast, in the middle cerebral artery, which is affected by hypoperfusion, BCCAo induces hypotrophic remodeling by a mechanism that involves a reduction of collagen I/III in association with increased MMP-1 and MMP-9 and consequent decrease in myogenic tone [51]. Therefore, it appears that the vascular changes seen in branches of the occluded vessels differ from those occurring in other vascular trees. The mechanisms involved in BCCAo-induced brain arteriogenesis are not fully understood, and the contribution of tissue ischemia to this process is not known. Notably, the phenomenon of vertebrobasilar arteriogenesis was homogeneous across animals, in strong contrast to the variable degree of acute ischemic damage, and this process was not detected in hypoperfused branches of the internal carotid artery. These findings are in agreement with the notion acquired in other vascular beds that, contrarily to angiogenesis, arteriogenesis is not induced by ischemia but results from the increased fluid shear stress derived from vessel occlusion [23]. Among the molecular determinants of arteriogenesis, expression of growth factors, adhesion molecules and chemokines, such as monocyte chemoattractant protein-1, in vascular smooth muscle cells have been identified as a critical factor for the initiation of arteriogenesis [56,57,58], together with monocyte recruitment to the arteries [59]. These effects promote vascular remodeling and are thought to be beneficial by restoring blood flow after arterial occlusion [60]. Understanding the molecular mechanisms underlying this process may lead to the development of therapeutic strategies to improve blood flow after chronic hypoperfusion.

In conclusion, our multimodal and longitudinal imaging approach to characterize the BCCAo model demonstrated *in vivo* a variable degree of acute ischemic lesions and delayed subcortical neurodegeneration, and also consistent white matter degeneration and arteriogenesis. We show that the BCCAo model is suitable for studying progressive brain degeneration caused by insufficient blood supply, taking into account that visually cued behavioral tasks might not be performed in correlation as a result of optic tract degeneration. In addition, we provide a new analytical method to assess physical alterations in main cerebral arteries *in vivo*. This approach could be used to monitor the development of structural cerebrovascular abnormalities and the effect of treatments that seek to facilitate vascular adaptive responses to chronic brain hypoperfusion. 

## Supporting Information

Figure S1 **Timeline of the followed experimental design.** Blue lines account for the behavioural tests. All animals performed the MRI protocol. A separate group underwent both MRI and behavioural testing (sham n= 7, BCCAo n=7). TOF: time of flight 3D angiography, T2: T2 relaxometry maps, MDEFT: Modified Driven Equilibrium Fourier Transform, DTI: diffusion tensor imaging, DSCE: dynamic susceptibility contrast enhanced imaging, ReD: Rediscrimination.
(TIFF)

Figure S2 **Vascular growth after BCCAO was calculated from the Maximal Intensity Projection (MIP) of the cropped base of the brain (A).** The ratio between the area occupied by the projected arteries and the area of the brain projection (B) was used to indirectly measure the vascular growth in Sham and BCCAo animals.
(TIFF)

Figure S3 **Dynamic susceptibility contrast imaging for evaluation of cerebral perfusion.** Regions of interest evaluated, from left to right, prefrontal coretx (pCx), caudate putamen (CP) and retrosplenial coretx (rCx). All Rois were drawn bilaterally and results are presented as the average ± SEM.
(TIFF)

Figure S4 **Representative photomicrographs of a confocal microscopic sections of basilar artery used to quantify the number of smooth muscle cells.** A) full ring; B) magnification of the window show in A. Natural autofluorescence of elastin (green) and nuclear staining (blue) are shown. AC, adventitial cell; EC, endothelial cell; SMC, smooth muscle cell, IEL internal elastic lamina.
(TIFF)

Figure S5 **Overlay of all lesions manifested by BCCAo rats 24h after surgery.** In 70% (9 out of 13) of the rats, BCCAo induced unilateral focal acute damage manifested as areas of increased T2 values located in the striatum with widely variable sizes ranging from 1.7 mm3 to 27.13 mm3.
(TIFF)

Figure S6 **Time course measurements of azygos-pericallosal and left and right middle cerebral arteries (MCA) before and after BCCAO.** Length (left graphs) and tortuosity index (right graphs) progression of azygos-pericallosal (A) and left and right middle cerebral arteries (B and C) were evaluated at 7 different time points, before and after BCCAO. White and black circles represent sham (n= 4) and BCCAO (n = 6) animals respectively.
(TIFF)

Figure S7 **Morphometric evaluation of middle cerebral artery (MCA) and collagen content quantification.** A) Vessel diameter. B) Lumen diameter. C) Cross-sectional area (CSA). D) Collagen I/III fluorescent intensity expressed in arbitrary units. White and black bars represent sham (n= 4) and BCCAO (n = 6) animals respectively.
(TIFF)

Table S1 **Two-way ANOVAs for length and tortuosity of the main craniocerebral arteries after bilateral common carotid artery occlusion assessed by time of flight (TOF) angiographic images.** Two-way analysis of variance (ANOVA), with treatment (BCCAo or sham operated animals) as the between factor and time (hours or days) as the within-subject factor. See material and methods for details.
(DOCX)

Table S2 **Two-way ANOVA for cerebral blood flow parameters measured by dynamic susceptibility contrast imaging.** ANOVA with treatment (BCCAo or sham operated animals) as the between factor and structure (caudate putamen, CP; prefrontal cortex, pCx; retrosplenial cortex, rCx) as the within-subject factor.
(DOCX)

Table S3 **Means and standard deviation (SD) of Fractional Anisotropy (FA) mean, axial and radial diffusivities (MD, AD, RD) for the main regions of significant changes obtained in the voxel-based analysis.** Cx, cortex.
(DOCX)
