# Supplementary figures and images for: The Ins and Outs of the BCCAo Model for Chronic Hypoperfusion: A Multimodal and Longitudinal MRI Approach
Source: PLoS One. 2013 Sep 18;8(9):e74631. doi: 10.1371/journal.pone.0074631 (PMC3776744; doi:10.1371/journal.pone.0074631)

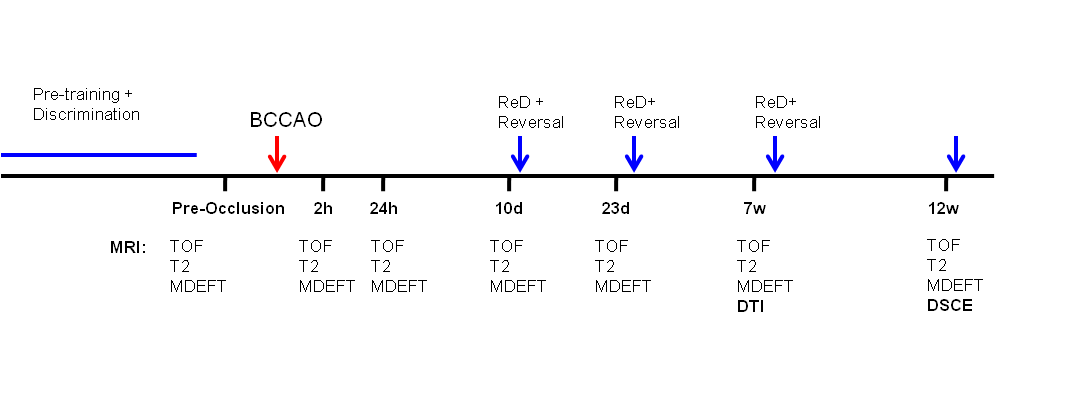

Supplement: Figure S1 — Timeline of the followed experimental design. Blue lines account for the behavioural tests. All animals performed the MRI protocol. A separate group underwent both MRI and behavioural testing (sham n= 7, BCCAo n=7). TOF: time of flight 3D angiography, T2: T2 relaxometry maps, MDEFT: Modified Driven Equilibrium Fourier Transform, DTI: diffusion tensor imaging, DSCE: dynamic susceptibility contrast enhanced imaging, ReD: Rediscrimination. (TIFF) [file pone.0074631.s001.tiff]

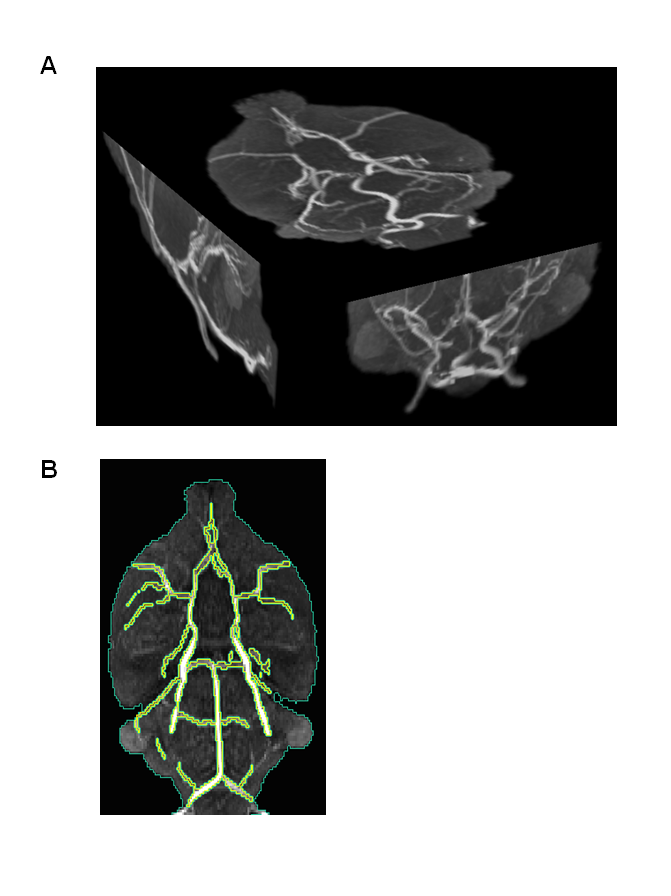

Supplement: Figure S2 — Vascular growth after BCCAO was calculated from the Maximal Intensity Projection (MIP) of the cropped base of the brain (A). The ratio between the area occupied by the projected arteries and the area of the brain projection (B) was used to indirectly measure the vascular growth in Sham and BCCAo animals. (TIFF) [file pone.0074631.s002.tiff]

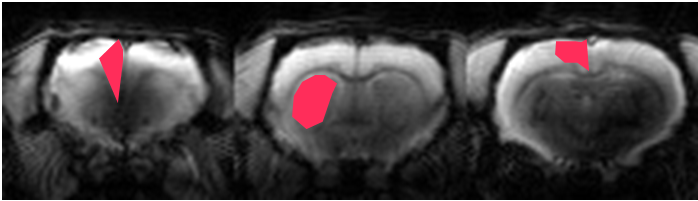

Supplement: Figure S3 — Dynamic susceptibility contrast imaging for evaluation of cerebral perfusion. Regions of interest evaluated, from left to right, prefrontal coretx (pCx), caudate putamen (CP) and retrosplenial coretx (rCx). All Rois were drawn bilaterally and results are presented as the average ± SEM. (TIFF) [file pone.0074631.s003.tiff]

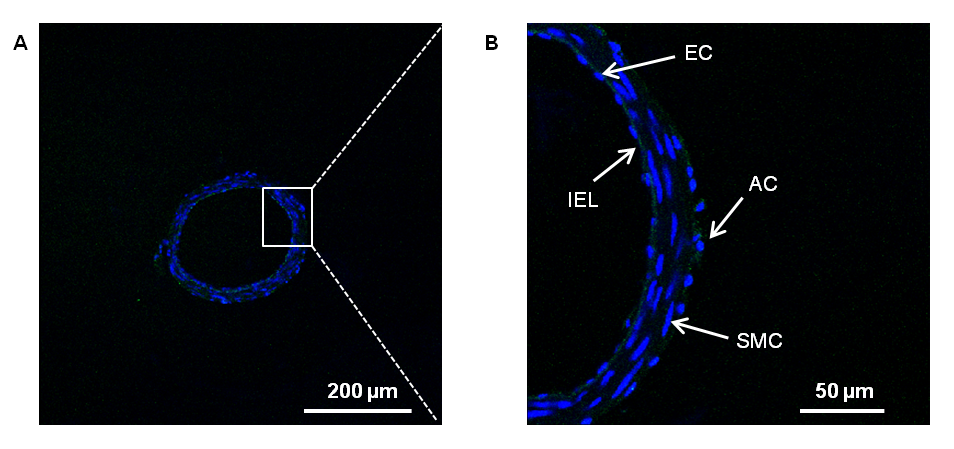

Supplement: Figure S4 — Representative photomicrographs of a confocal microscopic sections of basilar artery used to quantify the number of smooth muscle cells. A) full ring; B) magnification of the window show in A. Natural autofluorescence of elastin (green) and nuclear staining (blue) are shown. AC, adventitial cell; EC, endothelial cell; SMC, smooth muscle cell, IEL internal elastic lamina. (TIFF) [file pone.0074631.s004.tiff]

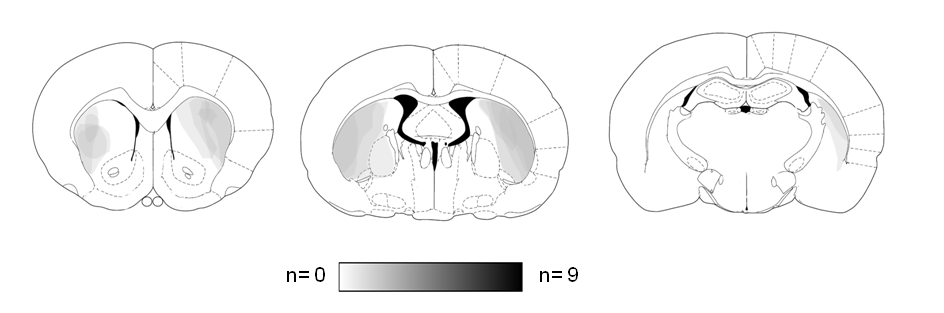

Supplement: Figure S5 — Overlay of all lesions manifested by BCCAo rats 24h after surgery. In 70% (9 out of 13) of the rats, BCCAo induced unilateral focal acute damage manifested as areas of increased T2 values located in the striatum with widely variable sizes ranging from 1.7 mm3 to 27.13 mm3. (TIFF) [file pone.0074631.s005.tiff]

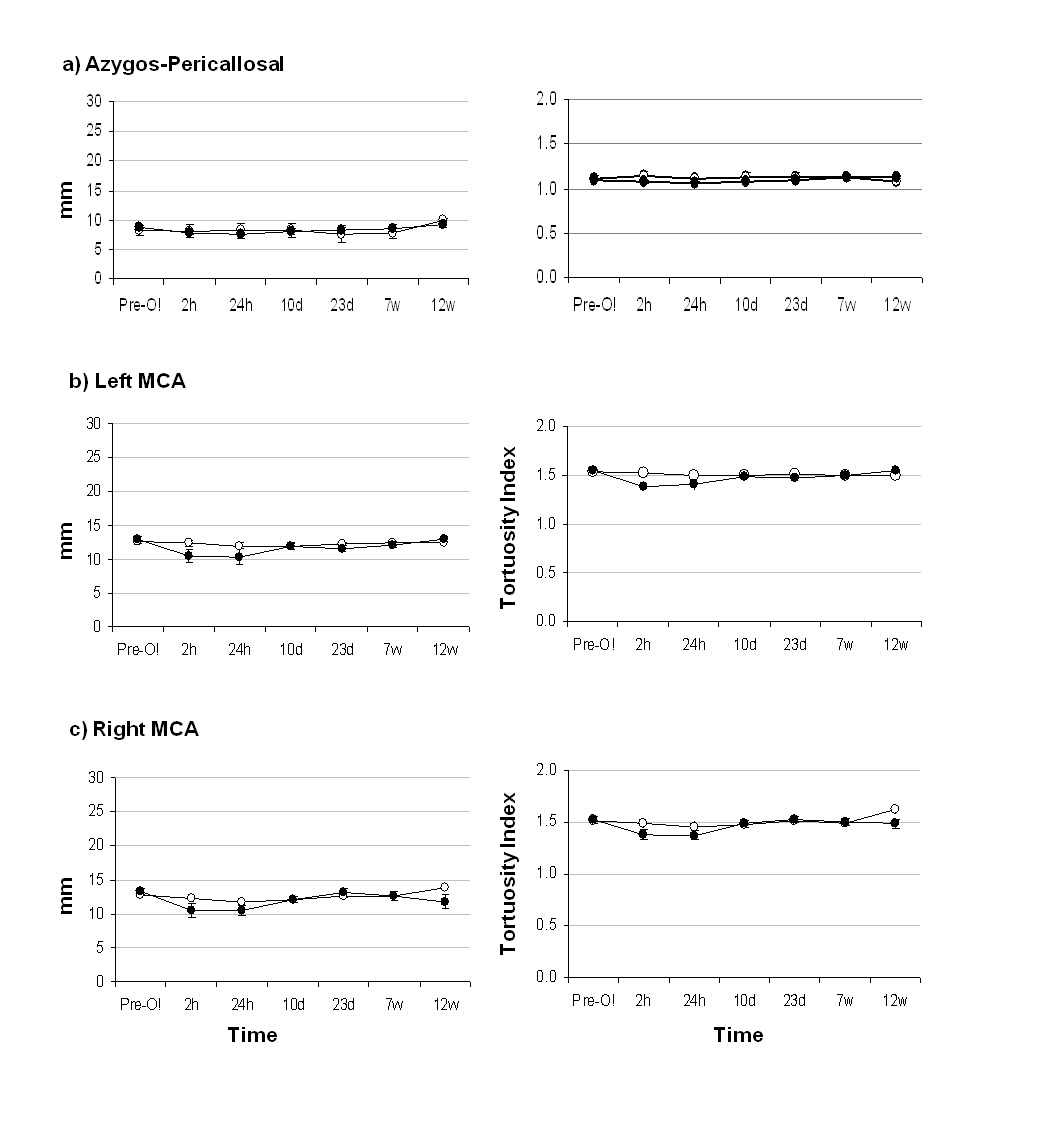

Supplement: Figure S6 — Time course measurements of azygos-pericallosal and left and right middle cerebral arteries (MCA) before and after BCCAO. Length (left graphs) and tortuosity index (right graphs) progression of azygos-pericallosal (A) and left and right middle cerebral arteries (B and C) were evaluated at 7 different time points, before and after BCCAO. White and black circles represent sham (n= 4) and BCCAO (n = 6) animals respectively. (TIFF) [file pone.0074631.s006.tiff]

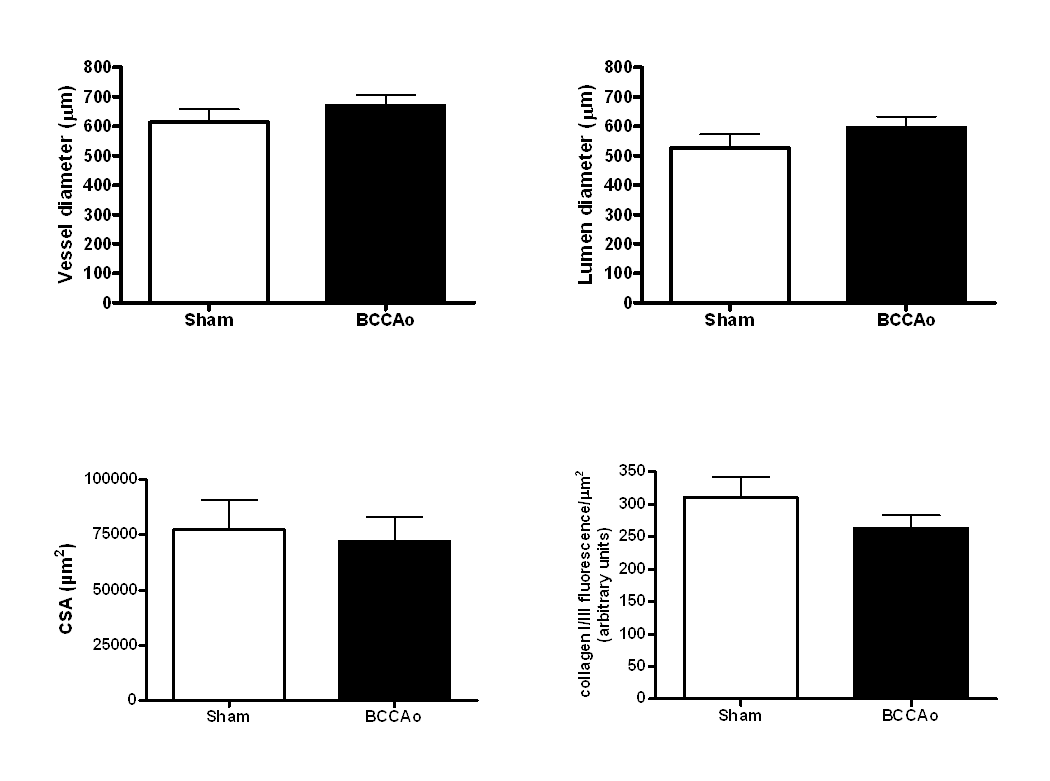

Supplement: Figure S7 — Morphometric evaluation of middle cerebral artery (MCA) and collagen content quantification. A) Vessel diameter. B) Lumen diameter. C) Cross-sectional area (CSA). D) Collagen I/III fluorescent intensity expressed in arbitrary units. White and black bars represent sham (n= 4) and BCCAO (n = 6) animals respectively. (TIFF) [file pone.0074631.s007.tiff]
