# Supplementary material for: The Ins and Outs of the BCCAo Model for Chronic Hypoperfusion: A Multimodal and Longitudinal MRI Approach
Source: PLoS One. 2013 Sep 18;8(9):e74631. doi: 10.1371/journal.pone.0074631 (PMC3776744; doi:10.1371/journal.pone.0074631)
Supplement: Table S1 — Two-way ANOVAs for length and tortuosity of the main craniocerebral arteries after bilateral common carotid artery occlusion assessed by time of flight (TOF) angiographic images. Two-way analysis of variance (ANOVA), with treatment (BCCAo or sham operated animals) as the between factor and time (hours or days) as the within-subject factor. See material and methods for details. (DOCX) [file pone.0074631.s008.docx]

|  | Vertebro-basilar system | | Right MCA | | Left MCA | | | Azygos-pericallosal | | |
| --- | --- | --- | --- | --- | --- | --- | --- | --- | --- | --- |
| **Length** | *F*-value | *P*-value | *F*-value | *P*-value | *F*-value | | *P*-value | *F*-value | *P*-value | |
| Time (T) | *F* _1,8_ = 4.677 | *n.s.* | *F* _1,8_ = 0.063 | *n.s.* | *F* _1,8_ = 0.128 | | *n.s.* | *F* _1,8_ = 0.095 | *n.s.* | |
| Treatment (Tt) | *F* _5,40_ = 13.304 | *P<* 0.001 | *F* _5,40_ = 2.200 | *n.s.* | *F* _5,40_ = 2.861 | | *P<* 0.05 | *F* _5,40_ = 0.642 | *n.s.* | |
| T x Tt | *F* _5,40_ = 9.821 | *P<* 0.001 | *F* _5,40_ = 1.614 | *n.s.* | *F* _5,40_ = 1.464 | | *n.s.* | *F* _5,40_ = 1.360 | *n.s.* | |
|  |  |  |  |  |  |  |  |  |  |  |
| **Tortuosity** |  |  |  |  |  |  |  |  |  |  |
| Time (T) | *F* _1,8_ = 32.749 | *P<* 0.001 | *F* _1,8_ = 0.079 | *n.s.* | *F* _1,8_ = 0.016 | *n.s.* | | *F* _1,8_ = 1.147 | *n.s.* |  |
| Treatment (Tt) | *F* _5,40_ = 16.160 | *P<* 0.001 | *F* _5,40_ = 2.015 | *n.s.* | *F* _5,40_ = 1.476 | *n.s.* | | *F* _5,40_ = 1.992 | *n.s.* |  |
| T x Tt | *F* _5,40_ = 16.295 | *P<* 0.001 | *F* _5,40_ = 1.107 | *n.s.* | *F* _5,40_ = 0.897 | *n.s.* | | *F* _5,40_ = 2.186 | *n.s.* |  |
