# Supplementary material for: The Ins and Outs of the BCCAo Model for Chronic Hypoperfusion: A Multimodal and Longitudinal MRI Approach
Source: PLoS One. 2013 Sep 18;8(9):e74631. doi: 10.1371/journal.pone.0074631 (PMC3776744; doi:10.1371/journal.pone.0074631)
Supplement: Table S2 — Two-way ANOVA for cerebral blood flow parameters measured by dynamic susceptibility contrast imaging. ANOVA with treatment (BCCAo or sham operated animals) as the between factor and structure (caudate putamen, CP; prefrontal cortex, pCx; retrosplenial cortex, rCx) as the within-subject factor. (DOCX) [file pone.0074631.s009.docx]

|  | Maximum | | Time to peak | | relCBV (AUC) | | FWHM | |
| --- | --- | --- | --- | --- | --- | --- | --- | --- |
|  | *F*-value | *P*-value | *F*-value | *P*-value | *F*-value | *P*-value | *F*-value | *P*-value |
| Structure (S) | *F* _2,54_ = 4.677 | *P<* 0.01 | *F* _2,54_ = 0.063 | *n.s.* | *F* _2,54_ = 0.128 | *n.s.* | *F* _2,54_ = 0.095 | *P<* 0.001 |
| Treatment (Tt) | *F* _1,54_ = 13.304 | *P<* 0.001 | *F* _1,54_ = 2.200 | *P<* 0.01 | *F* _1,54_ = 2.861 | *n.s.* | *F* _1,54_ = 0.642 | *P<* 0.001 |
| S x Tt | *F* _2,54_ = 9.821 | *n.s.* | *F* _2,54_ = 1.614 | *n.s.* | *F* _2,54_ = 1.464 | *n.s.* | *F* _2,54_ = 1.360 | *n.s.* |
