# Supplementary material for: The Ins and Outs of the BCCAo Model for Chronic Hypoperfusion: A Multimodal and Longitudinal MRI Approach
Source: PLoS One. 2013 Sep 18;8(9):e74631. doi: 10.1371/journal.pone.0074631 (PMC3776744; doi:10.1371/journal.pone.0074631)
Supplement: Table S3 — Means and standard deviation (SD) of Fractional Anisotropy (FA) mean, axial and radial diffusivities (MD, AD, RD) for the main regions of significant changes obtained in the voxel-based analysis. Cx, cortex. (DOCX) [file pone.0074631.s010.docx]

|  |  | control | | BCCAo | |
| --- | --- | --- | --- | --- | --- |
| FA | # Voxels | mean | SD | mean | SD |
| Piriform Cx | 47 | 0.271826 | 0.0423652 | 0.240394 | 0.0371281 |
| Optic nerve | 13 | 0.280593 | 0.0215184 | 0.234979 | 0.0306337 |
| Optic tract | 49 | 0.30783 | 0.044819 | 0.254775 | 0.0313038 |
| Prefrontal Cx | 37 | 0.235286 | 0.0525221 | 0.210733 | 0.0517871 |
| Thalamus | 164 | 0.251233 | 0.0253314 | 0.223116 | 0.02222 |
| Insular Cx | 23 | 0.319701 | 0.035354 | 0.283024 | 0.0317976 |
| Hipoccampus | 45 | 0.18531 | 0.02544 | 0.168832 | 0.0227486 |
| Lateral striatum | 41 | 0.199516 | 0.0222847 | 0.226009 | 0.0244141 |
|  |  |  |  |  |  |
| MD |  |  |  |  |  |
| Cingulum | 162 | 0.00068348 | 1.94E-05 | 0.00066395 | 3.36E-05 |
| Lateral striatum | 103 | 0.00061085 | 2.02E-05 | 0.00064922 | 2.73E-05 |
|  |  |  |  |  |  |
| RD |  |  |  |  |  |
| Cingulum | 124 | 0.000610107 | 2.87E+00 | 0.000597958 | 4.59E+00 |
| Lateral striatum | 79 | 0.000522436 | 1.54E+00 | 0.000541741 | 1.53E+00 |
|  |  |  |  |  |  |
| AD |  |  |  |  |  |
| Cingulum | 176 | 0.000867397 | 5.73E+00 | 0.000854007 | 7.70E+00 |
| Striatum | 108 | 0.000756146 | 3.08E+00 | 0.000784857 | 3.54E+00 |
| Thalamus | 60 | 0.000805562 | 3.55E+00 | 0.000779877 | 3.54E-01 |
